# Emergency cricothyroidotomy in difficult airway simulation – a national observational study of Air Ambulance crew performance

**DOI:** 10.1186/s12873-022-00624-6

**Published:** 2022-04-09

**Authors:** Åke Erling L. Andresen, Jo Kramer-Johansen, Thomas Kristiansen

**Affiliations:** 1 Department of Research, The Norwegian Air Ambulance Foundation, N-0184 Oslo, Norway; 2 Institute of Clinical Medicine, Faculty of Medicine, University of Oslo, Oslo, Norway; 3 Department of Anaesthesiology and Intensive Care, Drammen Hospital, Vestre Viken Hospital Trust, Drammen, Norway; 4 Department of Prehospital Services, Vestre Viken Hospital Trust, Drammen, Norway; 5 Norwegian Advisory Unit on Prehospital Emergency Medicine (NAKOS), Division of Prehospital Services, Oslo University Hospital, Oslo, Norway; 6 Department of Anaesthesiology and Critical Care Medicine, Division of Emergencies and Critical Care, Oslo University Hospital, Oslo, Norway

**Keywords:** Emergency medicine, Airway management, Prehospital, Simulation, Quality improvement

## Abstract

**Background:**

Advanced prehospital airway management includes complex procedures carried out in challenging environments, necessitating a high level of technical and non-technical skills. We aimed to describe Norwegian Air Ambulance-crews’ performance in a difficult airway scenario simulation, ending with a “cannot intubate, cannot oxygenate”-situation.

**Methods:**

The study describes Air Ambulance crews’ management of a simulated difficult airway scenario. We used video-observation to assess time expenditure according to pre-defined time intervals and technical and non-technical performance was evaluated according to a structured evaluation-form.

**Results:**

Thirty-six crews successfully completed the emergency cricothyroidotomy with mean procedural time 118 (SD: ±70) seconds. There was variation among the crews in terms of completed procedural steps, including preparation of equipment, patient- monitoring and management. The participants demonstrated uniform and appropriate situational awareness, and effective communication and resource utilization within the crews was evident.

**Conclusions:**

We found that Norwegian Air Ambulance crews managed a prehospital “cannot intubate, cannot oxygenate”-situation with an emergency cricothyroidotomy under stressful conditions with effective communication and resource utilization, and within a reasonable timeframe. Some discrepancies between standard operating procedures and performance are observed. Further studies to assess the impact of check lists on procedural aspects of airway management in the prehospital environment are warranted.

## Background

Airway management has the highest priority in emergency medicine, and basic airway management by opening and clearing airways is a core skill for all personnel in Emergency Medical Services (EMS). Successful prehospital airway management is associated with training and competence [1, 2]. EMS frequently deliver tiered care, reserving interventions requiring extensive experience and training to a limited cohort of providers. In Norway, this is a service provided by a prehospital anaesthesiologist, usually from the Air Ambulance Services [3].

Prehospital anaesthesia with endotracheal intubation (ETI) is an advanced high-risk procedure, and failure can lead up to a “Cannot Intubate, Cannot Oxygenate (CICO)”-situation [4]. A CICO-situation in this setting requires front-of-neck access, and a scalpel based, emergency cricothyroidotomy (EC) is the recommended procedure [2]. A prehospital observational study reported a surgical airway incidence of 1.2% among 7256 prehospital ETIs in a trauma population [5]. Thus, EC is a rare, but essential procedure, as it constitutes the last measure in difficult airway-algorithms [2, 3].

Prospective studies of EC in the prehospital environment may be impossible to perform. Simulation, however, offers the possibility to train and test skills for rare interventions, and provide clinical managers specific knowledge on advanced airway management quality, and crew performance [6]. In addition to technical skills, human factors and teamwork are major contributors to performance in high-risk work areas, such as prehospital EMS [7].

## Methods

### Aim

The aim of this study was to describe Air Ambulance-crews’ performance in a simulated difficult airway scenario, assessing technical performance of prehospital anaesthesia, emphasising the EC procedure, and non-technical aspects of management including communication, planning and team utilisation.

### Study design

The study was an observational trial of team performance in a simulation scenario, assessed by video, using predefined scoring of technical and non-technical skills and time intervals. The study is reported according to the STROBE-statement recommendations, including extensions for simulation-based research [8].

### Setting

Advanced prehospital airway-management in Norway is reserved for anaesthesiologists working in the EMS [3]. The main stem of this service consists of 13 Rotor Wing Air Ambulances (RW), seven Search and Rescue Helicopters (SAR) and five Rapid Response Car (RRC)-bases. The teams in the Rotor Wing Air Ambulances include additional staff with a Helicopter-EMS-crewmember (HCM) and a pilot [9]. The HCM is a nurse and/or paramedic, with a minimum of 2 years prehospital experience. The HCM is trained to assist the anaesthesiologist during medical procedures. RRCs include a Paramedic in addition to the anaesthesiologist. RRC with RW and SAR-teams are used when road access is faster and more convenient near air ambulance bases.

The study was conducted at a national Air Ambulance training facility, Camp Torpomoen. The camp is financed by The Norwegian Air Ambulance Foundation and invites personnel from all Air Ambulance Departments in Norway to lectures and simulated scenarios within medicine, rescue-operations, and flight operations. Participation in the study was voluntary, and participation in the training session was possible without being part of the study. Participation at the camp is mandatory for HCMs and pilots in the civil Air Ambulance, while anaesthesiologists from all services were invited to attend. One day prior to the trial all participants underwent theoretical and practical lectures on the learning objectives of the simulated scenario and advanced airway management, including front-of-neck EC with a bougie-assisted Rapid Four Step Technique (RFST) [10].

### Participants

All anaesthesiologists, HCMs’ and pilots attending the training sessions were eligible for inclusion (Table 1).

**Table 1:** Professional characteristics of the 36 attending crews

Crewmembers:	Professional characteristics:
Anaesthesiologist	• Consultant, or more than 4 years’ experience. • Prehospital training. • Working in Rotor Wing Air Ambulance, Rotor Wing Search and Rescue or Rapid Response Car.
HCM	• Authorised as health personnel, nurse or paramedic. • Experienced from ambulance service. • Trained in rescue operations and as assistant to medical doctor on ground and to pilot in-flight. The roles are specific to the Norwegian Air Ambulance concept.
Pilot	• Extensive experience from flight operations. • Minimal formal medical training.

### Test scenario

Each crew was presented an identical scenario where they should do a primary response to assist Paramedics with a patient located in a parked ambulance. The clinical details are described in Table 2.

**Table 2:** Description of the simulation scenario: Study Model, anamnestic details, facilitator instructions, equipment and learning objectives

Study Model	• Adult patient simulator^a^ with advanced airway options. • The manikin was built up with a large thorax and a thick neck, as in adipositas and goitre, and put in a “cannot intubate”-modus. • After the first skin incision, the facilitator emptied a 10 cc syringe with theatre-blood in the field. • Vital signs and values (Blood pressure (BP), oxygen saturation (SpO_2_), heart rate (HR), 3-lead electrocardiogram (ECG), End-tidal CO_2_) given remotely^b^ to the patient monitor when accomplished.
Anamnestic details	• A paramedic-manned ambulance is requesting Air Ambulance for assistance. • The patient is a 60-year-old female, obese with an un-operated goitre and a history of breathing-problems and reduced general health for the last week. She is in respiratory distress, aggravated in the last hours.
Instruction for facilitators:	• Initial physiological status: GCS 13 points, SpO_2_ = 85%, SBP 105 mmHg, HR = 110 / min. • After appropriate first intervention (positioning and supplemental oxygen) transient improvement. • Ultimately, patient deteriorates with falling SpO_2_, followed by decreasing GCS, forcing the crew to attempt an RSI. The manikin was put in a “cannot intubate”-modus, forcing the team to perform an EC.
Equipment	• Advanced Life Support-Ambulance. • Emergency bag equal to standard national Air Ambulance-leve l[11]. • Equipment for surgical airway including: Scalpel, tracheal hook, Cuffed 6.0 mm endotracheal tube and a Frova Intubating Introducer® (Cook Medical, USA).
Learning objectives	• Identify a difficult airway • Ensure adequate monitoring, preparations and conduction of RSI. • Solve CICO with an EC

^a^Lærdal SimMan 3G, Lærdal Foundation, Norway

^b^SimMon, Castle+Andersen Aps, Denmark

The participants were encouraged to manage the patient during simulation as in a real mission.

The scenario was led by senior facilitators with extensive experience from Air Ambulance clinical service and simulation teaching. The facilitators ran the simulation based on a written manual with pre-determined responses to treatment options and the study objectives. Manikin and monitors were operated remotely by an assistant (Table 2).

The scenario progressed in a stepwise manner, in order to let all participants face the learning objectives (Table 2).

### Data collection and study variables

The scenario was filmed with a camera placed in the back of the ambulance providing a clear view to the scenario. Our data material consists of video-material of the crews’ communications and actions during the simulation. To reduce bias and to quantify performance in an advanced clinical scenario, predefined time intervals, binary quality indicators of procedural steps, and quality indicators of non-technical skills were extrapolated from the video-material. These were based on current literature and the Standard Operating Procedure, Oslo University Hospital, Air Ambulance Department [2, 3, 12–14].

**Fig. 1:**
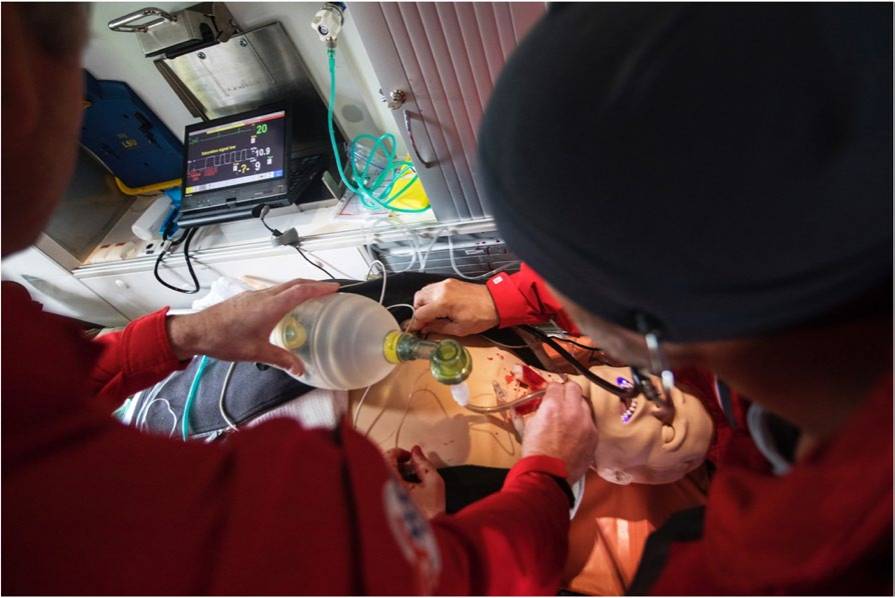
Doctor and HCM from the Air Ambulance working on the study model

Success was defined as being able to do an EC and perform an ETI through the cricothyroid membrane and start ventilation with a self-inflatable bag (Fig. 1).

Time intervals were measured from (1) decision to do a surgical airway, and (2) the first skin incision with the scalpel. Stop-point was the first successful ventilation with a self-inflatable bag on the tube successfully placed in the trachea on the manikin.

The Anaesthetists non-technical skills (ANTS) -system is a validated approach to evaluate the non-technical aspects of crew performance [14, 15]. We developed a structured list of performance observations which could be scored with three possible variables (Yes/No/Partially). These points covered the four ANTS-categories: Situation awareness, team working, task management and decision making.

### Statistical methods

Performance indicators are presented as numbers and percentages. Time intervals are stated in seconds with mean values, standard deviation and range.

## Results

All 36 eligible Air Ambulance crews attending the camp participated in the study. The crews represented all Air Ambulance bases in Norway, including three doctors from the Air Force SAR-Helicopter and four doctors from the RRC, Oslo University Hospital (Table 1).

All crews successfully conducted the EC achieving ventilation with the bougie-assisted RFST-technique. The mean time interval for the procedure from first incision to ventilation was 118 s (SD: ±70, range: 40-322), and the duration from doctor’s decision to perform an EC to successful ventilation was 153 s (SD: ±80 s, range: 69-369).

Completed procedural steps by the crews with respect to monitoring, equipment and management are given in Table 3. Almost all crews established basic monitoring with pulse oximetry and non-invasive blood pressure, while less than one in four crews established three-lead electrocardiogram monitoring. Five crews prioritized invasive blood pressure monitoring. Two-thirds ensured that equipment for suction was present, while only 56% tested that the equipment was working. Almost all crews established a running intravenous line with crystalloids, but very few gave a fluid bolus prior to first Rapid Sequence Induction (RSI) attempt. Similarly, only four crews administered vasoactive medication prior to the RSI procedure. Less than half of the crews positioned the neck in “sniffing position” before RSI, while 50% palpated the cricothyroid membrane prior to induction. After failed ETI-placement, 92% of all crews initiated assisted ventilation with a self-expendable ventilation bag. Half of the crews then attempted a supraglottic airway device (SGA), while the other half decided to go straight to an EC. Among the crews who briefed a plan B consisting of a SGA (Table 3), 58% used the SGA. For the crews who did not brief, or only partly briefed, a plan B, 33% attempted a SGA.

**Table 3:** Procedural steps performed by crews stated in absolute numbers and percentages

STEPS:	Performed n (%)	Not performed n (%)	COMMENT / DESCRIPTION
EQUIPMENT			
Pulse oximetry	34 (94)	2 (6)	Monitoring with pulse oximetry initiated before RSI
Three-lead ECG	8 (22)	28 (78)	Monitoring with three-lead ECG initiated before RSI
Non-invasive blood pressure (NIBP)	35 (97)	1 (3)	Monitoring with NIBP initiated before RSI
Invasive blood pressure (IBP)	5 (14)	31 (86)	Monitoring with IBP initiated before RSI
Intravenous fluid	35 (97)	1 (3)	Establishing intravenous infusion with crystalloid
Additional intravenous route	13 (36)	23 (64)	Placement of extra peripheral venous cannula
Oxygen present	32 (89)	4 (11)	Presence of oxygen tank addresses loudly by one crewmember
Suction present and tested	20 (56)	16 (44)	Presence of suction addressed loudly by one crewmember, and functional testing applied
Preparation of equipment	15 (41)	21 (59)	Complete planning and preparing for additional airway equipment before RSI
PROCESS RELATED			
Optimisation of posture	24 (67)	12 (33)	Raising back of ambulance-stretcher when arriving to patient
Preoxygenation	35 (97)	1 (3)	Preoxygenation before conduction of RSI
Early assisted ventilation	16 (44)	20 (56)	Bag-valve-mask ventilation initiated first 2 min
Fluid bolus	3 (8)	33 (92)	Deliberately increasing intravascular volume before RSI
Vasoactive bolus	4 (11)	32 (89)	Deliberately increasing vascular resistance before RSI
Patient elevated head	14 (39)	22 (61)	Establishing the patient in “sniffing position” with an elevated thorax, suitable for RSI of adipose patient.
Assisted ventilation after failed RSI	34 (94)	2 (6)	Provide oxygen to patient by assisted ventilation with bag-mask-valve
Implementation of plan B	18 (50)	18 (50)	Use of supraglottic device after failed RSI
Doctor placed lateral to patient for RFST	34 (94)	2 (6)	Taking position at side of patients’ neck, opposed to standing behind head before RFST
Active extension of neck	15 (42)	21 (58)	Adequate optimization of patient before RFST
Active build-up under shoulders	6 (17)	30 (83)	Adequate optimization of patient before RFST
Doctor preparing medication	2 (6)	34 (94)	When not performed, HCM or pilot is preparing the RSI medications.
Early capnography	19 (53)	17 (47)	Connecting capnography first 10 s after ETT-placement
Auscultation	36 (100)	0	Bilateral auscultation to confirm ETT-placement

Prior to the EC procedure, the majority failed to elevate and extend the neck for optimised access to the cricothyriod membrane. Post-intubation, all crews assessed tube placement by auscultation, while half of the crews promptly used capnography for verifying tube placement.

The evaluation of non-technical skills is provided in Table 4. The crews displayed high situational awareness in terms of collecting adequate anamnestic information and recognition of a difficult airway situation. Closed-loop communication was widely employed, and available resources utilised with the majority of crewmembers partaking in the medical management; the HCM was participating in clinical decision-making and treatment in all cases. The pilots were actively involved in the scenario, e.g. with equipment assistance, in 28 (78%) of the simulations. Further, in one third of the cases, the pilots’ contribution played a major role in problem-solving, e.g.: the pilot being the first crewmember to address difficult airway, preparing difficult airway equipment, or taking initiative to improve positioning of the patient to facilitate the airway procedure.

**Table 4:** Non-technical skills demonstrated by crews during simulation

Non-technical skills	Performed n (%)	Not performed n (%)	Partially performed n (%)	COMMENT / DESCRIPTION
Situation awareness				
Anamnesis	34 (94)	1 (3)	1 (3)	Obtaining adequate anamnestic details from paramedic on-scene
Recognition of difficult airway	34 (94)	2 (6)	Not applicable	Possible difficult airway addressed loudly by one crewmember
Team working				
HCM involvement	36 (100)	0	0	HCM actively participating in assessment, treatment and use of equipment
Pilot involvement	28 (78)	8 (22)	0	Pilot participating in assessment, preparation or treatment.
Pilot major contribution	11 (33)	22 (66)	Not applicable	Pilot actively and independently contributing to assessment, preparation or treatment.
Task management				
Brief RSI medications	33 (92)	2 (6)	1 (3)	Giving a concise brief on which medications and dosage before RSI
Brief Plan B for alternative airway	25 (69)	8 (22)	3 (9)	Supraglottic airway device if RSI-failure.
Brief Plan C for alternative airway	11 (30)	14 (39)	11 (31)	Surgical front of neck-access if RSI-failure
Decision making				
Closed-loop communication	33 (92)	3 (8)	0	Deliberated use of closed loop in team communication
Checklist	1 (3)	35 (97)	0	Use of standardized checklist before RSI

The crew resource utilisation was also evident by the fact that in 34 of 36 crews, other crewmembers than the doctor were preparing the medication (Table 3). A full “double-signature” check of medications was subsequently performed.

At induction of anaesthesia, the majority did an RSI-brief, and included an alternative airway strategy with the use of a supraglottic airway device. Only one third of these briefs included a plan for EC.

Only one crew used a structured checklist during the scenario.

## Discussion

Our study shows that in a high-fidelity simulation with a manikin model, Norwegian Air Ambulance crews managed to solve a difficult airway situation successful and in a timely manner. Some heterogeneity is observed in the technical conduct of the scenarios, but effective team cooperation and task management characterise the crews’ performance.

The high success rate is in accordance with clinical data from a previous large observational study [5]. Procedural duration for RFST was 118 s, with maximum time 322 s.

Experimental studies have found time expenditure for RFST-procedure in a range about 60 s [16–18]. These studies have in common that they have been performed on various laryngeal models, in which the operator does not have to consider other clinical factors. Often the procedures have been performed by a single provider without assistance, to allow for comparison of different techniques. Such experimental settings may not be completely generalisable to the clinical setting as the stress of a CICO-situation may not have been reproduced.

In this setting another important time interval is from decision to do EC, until completion. A mean duration of 159 s is in our opinion both realistic and an acceptable result, indicating that most crews were prepared to convert from an endotracheal intubation procedure to an EC procedure.

A standardised RSI-protocol is advised in emergency medicine [19]. Protocol briefing with alternative airway management plans before problems arise ensures that the entire crew is prepared and knows their role in a critical situation like CICO, in order to shorten the hypoxic time interval [20]. Air Ambulance crews who fulfil these requirements can start the EC more immediately when the attempted endotracheal intubation must be aborted. Failure to optimize patient position, lack of thorough brief of alternative airway plans and differences in preparation of equipment can be contributing factors to long procedural duration in some crews, leading to a prolonged hypoxemia for the patient.

Prehospital advanced airway management is a complex medical procedure. The majority of the learning objectives are fulfilled by all crews. Recognition of a potential difficult airway was stated early, yet only half of the doctors palpated the neck anatomy before induction of anaesthesia. We found that RSI-preparation was acceptable, and the majority followed the predetermined objectives. Important medical treatment was provided with little delay. Our findings indicate that there are room for improvement regarding preparations. The Plan B with a supraglottic device is only carried out by 50%. We cannot determine if the other crews forget to follow their plan, or if they find the clinical situation so critical that they decide to go for the definitive airway solution with an EC without trying out other steps. The results imply that crews with a clear brief of an alternative airway strategy, is more likely to follow the difficult airway algorithm.

Correct positioning of the patient before RSI and re-positioning to achieve elevation of the neck before EC, improves success rates. Extension of the neck is recommended before the EC procedure to ease passing of the endotracheal tube (ETT) [21]. Even if the RFST was carried out correctly, some crews experienced difficulties when they were ready to pass the ETT through the membrane.

ETI must be confirmed with end-tidal CO_2_, and this is well known to Air Ambulance-providers [2]. Our study shows that this can be forgotten in a stressful setting. The use of checklists was almost non-existing. The true value of checklists for experienced anaesthesiologists can be debated. A recent meta-analysis indicates that there is no association between checklists and better clinical outcome [22]. On the other hand, implementation of checklists has been advocated in the literature, and it can be argued that stricter adherence to standard operating procedures can optimise the advanced prehospital airway management [23]. In a recent study in the Nordic countries 60.5% of anaesthesiologists used a RSI-checklist, but there was no difference in overall success rate [24]. Our results indicate that there is variation in airway management, and this is observed parallel to a very limited use of checklists.

Identifying treatment options and selecting airway management was also indicating that the Air Ambulance crews have a good situational awareness. The Crew Resource Management (CRM)-concept is derived from aviation and is designed to reduce human errors by using safety-management principles and training interventions [25]. The field of anaesthesiology was the first to adapt these principles in medicine. We also found the ANTS-principles to be a useful tool in this simulation study. Our study indicate that CRM is well incorporated in Norwegian H-EMS; e.g., there was extensive use of closed-loop communication and duplication checking. The HCM was supportive and assisted the doctor to a great extent in all simulations. We also found that the pilot was contributing substantially, and that their contribution was crucial in almost one third of the cases. Despite no formal education in emergency medicine, the pilots’ clinical understanding of the situation was higher than what may be expected. We observed that when the workload was high for the physician and HCM, the pilots took the role of the qualified assistant, and was able to provide important inputs to his colleagues. This implies high utilisation of the available resources. A previous study of Norwegian H-EMS reported need for improvement in simulation training and non-technical skills [26]. Our findings correspond better with several more recent studies that imply a shift towards increased focus on these important aspects of emergency medical care [27, 28].

### Limitations

This is an experimental setting with obvious lack of realism and feeling of lives at stake, and performance may be different in real life. Medical simulation is to a certain degree a realistic proxy for real emergency situations, and its use is supported in literature [29]. It has been claimed that when experienced anaesthesiologists struggle with medical emergencies in simulation it also indicates suboptimal real life patient care [30].

All participants attended a lecture and practical training the day before the scenario. It is likely that longer interval between training and testing, would result in poorer performance.

A clinical scenario with different participants and facilitators will have different group dynamics and there will be variation in performance, both regarding preparations and treatment. Thus, a limitation of this study is the human aspect in the difference in facilitators’ feedback and case progression during simulation. The clinical course of the case was directed by the manual, but at the facilitators’ discretion. Despite efforts to standardise the interventions, advanced medical simulation with different crews is complex and it is not possible to reproduce identical clinical trajectories. This may be a contributing factor to some of the observed discrepancies in the airway management. A prehospital CICO-situation requires a lot from the crew regarding planning, decision making and implementation of different airway strategies. A delay in any of these phases will result in prolonged hypoxia for the patient in a CICO situation. We were not able to capture the individual contribution of all these elements in terms of time expenditure in this study. EC procedural time was, however, in our opinion an objective and relevant indicator for quality of airway management in this simulated setting.

## Conclusion

The study shows that Norwegian Air Ambulance crews manage to solve a prehospital CICO-situation with an EC under stressful conditions within a reasonable timeframe. We observed a high level of performance regarding both technical- and non-technical skills among the crews. Effective communication and teamwork, utilising all crew resources, characterise the scenarios. Comparison with our predetermined objectives also disclosed some discrepancies with a substantial proportion of the crews not addressing key steps of monitoring and preparation. The use of a structured checklist was almost non-existing. How this relates the observed discrepancies is yet to be determined. We recommend future studies that assess the use of checklists and how they affect both time expenditure and compliance to standard operating procedures in similar scenarios.

## Data Availability

The dataset extracted from the video material regarding the pre-defined observational points is provided in a separate file. The video material is not published due to privacy considerations for the participants. The video material is stored on the research server at Vestre Viken Hospital Trust, and insight can be considered by the authors on reasonable request.

## Abbreviations

EMS: Emergency Medical Services
ETI: Endotracheal Intubation
CICO: Cannot Intubate, Cannot Oxygenate
RW: Rotor Wing
SAR: Search And Rescue
RRC: Rapid Response Car
HCM: Helicopter-EMS-crewmember
RFST: Rapid Four Step Technique
ANTS: The Anaesthetists Non-Technical Skills
RSI: Rapid Sequence Induction
ETT: Endotracheal Tube
SAG: Supraglottic Airway Device
CRM: Crew Resource Management
